# Findings of uncertain significance by optical coherence tomography (OCT) as prognostic factors in neovascular age-related macular degeneration (nAMD) treated with ranibizumab

**DOI:** 10.1186/s40942-022-00379-z

**Published:** 2022-04-21

**Authors:** Ricardo Hayashi-Mercado, Carla Pérez-Montaño, Jaime Reyes-Sánchez, Abel Ramírez-Estudillo

**Affiliations:** 1 Retina and Vitreous Department, Fundación Hospital Nuestra Señora de La Luz, Ezequiel Montes 135, 06030 Mexico City, Mexico; 2 Department of Population and Publica Health Sciences, Keck School of Medicine, University of Southern California, 1975 Zonal Ave, 90033 Los Angeles, CA USA

**Keywords:** OCT, Biomarker, nAMD, Ranibizumab

## Abstract

**Background:**

Biomarkers hold great promise for personalized medicine as information gained from diagnostic or progression markers can be used to tailor treatment to the individual for highly effective intervention in the disease process.

**Methods:**

The aim of this retrospective study was to evaluate the association between visual outcome and the presence of findings of uncertain significance by optical coherence tomography (OCT) pre and post loading dose in patients with neovascular age-related macular degeneration (nAMD) treated with ranibizumab.

**Results:**

Univariate analysis revealed a higher letter gain in those with presence of onion sign (+ 5.6 ETDRS letters, p = 0.04) absence of prechoroidal cleft (+ 3.7 ETDRS letters, p = 0.04), intraretinal pseudocysts (+4.8 ETDRS letters, p = 0.002), subretinal pseudocysts (+ 4.6 ETDRS letters, p = 0.005) and choroidal caverns (+ 4.4 ETDRS, letters p = 0.0065).

**Conclusions:**

The presence of prechoroidal cleft, intraretinal and subretinal pseudocysts and choroidal caverns were associated with lower visual gains. Moreover, we found that the onion sign is related as a biomarker of good prognostics.

*Trial registration* Registration number: 2021R13B2. Date of registration: 01/05/2020

## Background

Age-related macular degeneration (AMD) is the leading cause of legal blindness in elderly people, especially in developed countries [1]. In 2020, about 200 million people were affected by AMD worldwide, and the incidence is constantly increasing as a consequence of exponential population aging [2]. Neovascular AMD (nAMD) represents a small subset (less than 10%) of total AMD cases; however, the neovascular form is responsible for the majority of cases of severe visual loss in eyes with AMD [3].

“Biomarker” refers to a broad subcategory of medical signs that objectively indicate the state of health, and well-being of an individual. In clinical practice, they are useful in refinement of diagnosis, measuring disease progression or predicting and monitoring effects of therapeutic interventions. Biomarkers hold great promise for personalized medicine as information gained from diagnostic or progression markers can be used to tailor treatment to the individual for highly effective intervention in the disease process [4, 5].

Optical coherence tomography (OCT) is a reliable, quick, sensitive, non-invasive, user-friendly device that provides high-resolution in vivo imaging of retinal microstructures. These modalities aid in diagnosis of AMD, help in treatment decisions, identify the disease recurrence, and establish the visual prognosis [6, 7].

Literature search shows multiple signs based on cross-sectional OCT scans that are suggestive of choroidal neovascularization (CNV) in nAMD eyes [6–8]. The evaluation of these criteria that include presence of intraretinal or subretinal fluid, ill-defined boundaries of the neovascular lesion, and increase of central macular thickness (CMT) provides information about disease activity for retreatment decisions [8, 9]; however, not all structural changes on OCT suggest either active CNV or exudation. These signs are multifactorial in origin, including degenerative changes (pseudocysts, outer retinal tubulation), unique choroidal features directly associated with type 1 CNV, and retinal angiomatous proliferation lesions (prechoroidal clefts) or atrophic changes (choroidal caverns) [10].

In this study we define “findings of uncertain significance” as a group of biomarkers that have not been associated with a prognostic value on nAMD (onion sign, prechoroidal cleft, intraretinal and subretinal pseudocyst and choroidal caverns). The aim of this retrospective study was to evaluate the association between visual outcome and the presence of these findings of uncertain significance by OCT in patients with nAMD treated with ranibizumab.

## Methods

In this retrospective, cross-sectional and descriptive study, records of patients with a clinical diagnosis of treatment-naïve nAMD at Retina and Vitreous Department of Hospital de la Luz, Mexico City who were initiated a loading dose (3 monthly injections) of ranibizumab between January 2020 and August 2021 were reviewed for demographic information, OCT findings of uncertain significance, and the clinical outcomes of best corrected visual acuity (BCVA) and CMT. The study was approved by local institutional review board, and Human research was conducted according to the Tenets of the Declaration of Helsinki.

### Inclusion and exclusion criteria

Subjects were included in the study if the fulfilled the following criteria: (1) men or women ≥ 50 years, (2) CNV type 1 or 2 secondary to nAMD involving the central fovea, (3) BCVA 5–85 Early treatment diabetic retinopathy study (ETDRS) letters (20/40–20/800 Snellen equivalent), (4) had received a loading dose with 3 monthly injections of ranibizumab 0.5 mg/0.05 mL and (5) had OCT with a quality ≥ 20. Subjects were excluded from the study if they had any of the following: (1) history of vitrectomy, (2) previous antiangiogenic therapy, (3) uncontrolled systemic arterial hypertension and (4) cataract ≥ NC 2.5/NO 2.5/C 2.5/P 1 according to LOCS III [11].

### Spectral domain optical coherence tomography (SD-OCT) evaluation

SD-OCT scans of the macular region captured using Heidelberg Spectralis (Heidelberg Engineering, Heidelberg, Germany) within 1 month of the confirmed diagnosis of nAMD with central foveal involvement were included in the study. The analysis of images was performed at the central and the first 5 images of the macular region. The evaluations were performed independently by RHM and ARE. Any discrepancies were resolved with mutual discussion about the case.

### Findings of uncertain significance

**Fig. 1:**
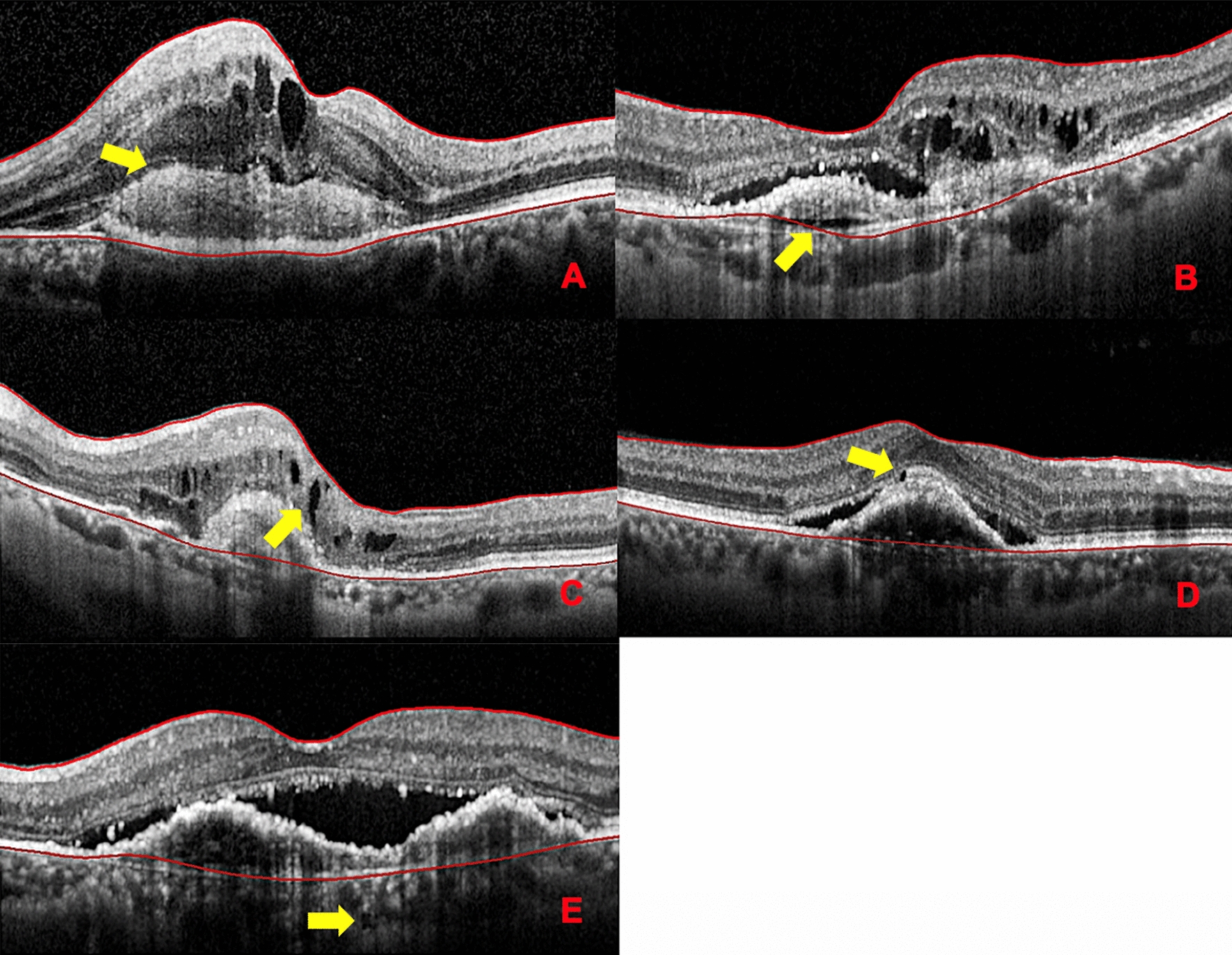
Cross-sectional optical coherence tomography of findings of uncertain significance (yellow arrow). **A** Onion sign. **B** Prechoroidal cleft. **C** Intraretinal pseudocyst. **D** Subretinal pseudocyst. **E** Choroidal cavern

Onion sign is a finding seen between retinal pigment epithelium (RPE) and Bruch membrane in the form of multilayered hyperreflective bands similar to multiple layers seen in an onion [12]. Prechoroidal cleft are defined as hyporeflective spaces sandwiched between two hyperreflective lines, the RPE, and Bruch membrane and are characterized by posterior bowing of Bruch membrane [13]. Intraretinal and subretinal pseudocyst are optically empty spaces without the presence of any hyperreflectivity at their borders with at least one concave or straight border [14, 15]. Choroidal caverns are a choroidal hyporeflective spaces with absence of hyperreflective border and hyporeflective lumen with a tail of hypertrasmission [16] (Fig. 1).

### Outcome measures

The clinical outcomes that were evaluated were BCVA and CMT. Both were measured pre and 1-month post-treatment.

### Statistics

Descriptive analysis was performed for each variable, and distributions assessed for departure from normality using Kolmogorov–Smirnov tests. The Pearson’s correlation coefficient was used to evaluate associations among the outcomes, and paired t-tests and Wilcoxon rank sum tests were used to evaluate changes in clinical outcomes pre and post-treatment depending on the presence or absence of each of the five findings of uncertain significance by OCT. All *p-*values (p) are 2-sided, and p < 0.05 was considered statistically significant. Statistical analysis was performed with STATA version 15.0 (Stata Corp LP, Texas, USA).

## Results

A total of 83 eyes of 83 patients were included in the analysis. Mean age of the participants was 77 ± 8.36 years, and most eyes were female 47 (56.6%). Mean (± standard deviation (SD)) baseline BCVA was 29.76 (± 22.66) ETDRS letters, post-treatment BCVA was 33.4 (± 24.24), with a mean difference between pre and post-treatment of 3.67 (± 13.68) ETDRS letters. Mean basal CMT was 468 (± 223.2) μm and mean post-treatment CMT 377.4 (± 216.55) μm with a mean difference of −90.79 (± 216.55) μm.

**Fig. 2:**
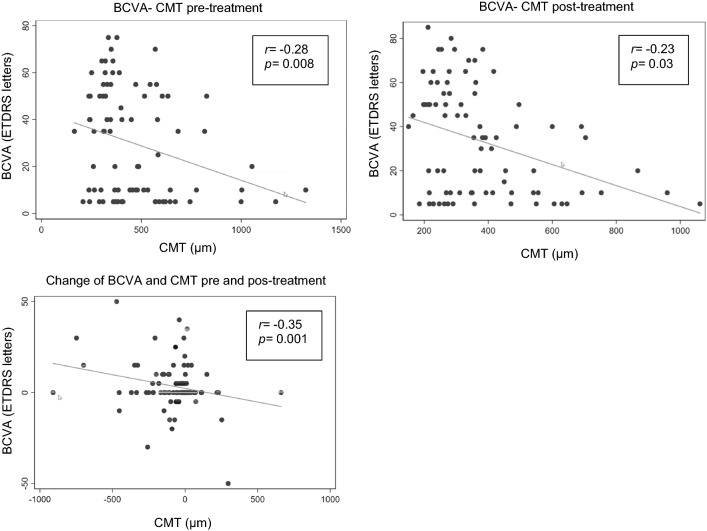
Scatter plot of BCVA vs CMT with linear regression. Pearson correlation coefficient (*r*) with significance (*p* value) is presented in the boxes

There was a statistically significant negatively correlation between BCVA and CMT basal (r = − 0.28, p = 0.008), BCVA and CMT post-treatment (r = − 0.23, p = 0.03), and between the change of BCVA and CMT pre and post-treatment (r = − 0.35, p = 0.001) (Fig. 2).

The frequencies of OCT findings of uncertain significance were analyzed by age and sex. Although mean age was similar in patients with or without each of the five findings of uncertain significance, prechoroidal cleft was found in a higher proportion of females (29.8%) than males (11.1%), and this association was statistically significant (p = 0.04) (Table 1).

**Table 1:** Findings of uncertain significance, age, gender and p value

Biomarker	Frequency (number of eyes)	Mean age with biomarker	Mean age without biomarker	p value*	Female (%)	Male (%)	p value**
Onion sign	32	76.4 ± 9	77.4 ± 8	0.58	34	44.4	0.33
Prechoroidal cleft	18	77.3 ± 8	77 ± 8	0.86	29.8	11.1	0.04
Intraretinal pseudocyst	32	78.9 ± 8	75.9 ± 8	0.11	34	44.4	0.33
Subretinal pseudocyst	16	76.2 ± 9	77.2 ± 8	0.65	19.2	19.4	0.97
Choroidal caverns	17	78.2 ± 9	76.7 ± 8	0.53	21.3	19.4	0.83

Comparison between the presence or absence of biomarkers according to age and gender

*Two–samples *t*-test with equal variance

**Pearson Chi^2^

Paired *t*-tests for comparing BCVA pre and post-treatment revealed a statistically significant letter gain in those with presence of onion sign (+ 5.6 ETDRS letters, p = 0.04), absence of prechoroidal cleft (+ 3.7 ETDRS letters, p = 0.04), absence of intraretinal pseudocysts (+ 4.8 ETDRS letters, p = 0.002), absence of subretinal pseudocysts (+ 4.6 ETDRS letters, p = 0.005), and absence of choroidal caverns (+ 4.4 ETDRS letters, p = 0.0065) (Table 2).

**Table 2:** Univariate analysis compared BCVA and findings of uncertain significance

	Eyes per group	Mean basal BCVA in ETDRS letters	Mean BCVA after loading dose in ETDRS letters	Difference in ETDRS letters	p value*
Without onion sign	51	33.1	35.6	+ 2.5	0.16
With onion sign	32	24.4	30	+ 5.6	0.04
Without prechoroidal cleft	65	32.9	36.6	+ 3.7	0.04
With prechoroidal cleft	18	18.3	21.9	+ 3.6	0.15
Without intraretinal pseudocyst	51	30.4	35.2	+ 4.8	0.002
With intraretinal pseudocyst	32	28.8	30.6	+ 1.8	0.54
Without subretinal pseudocyst	67	31.6	36.2	+ 4.6	0.005
With subretinal pseudocyst	16	21.9	21.9	0	1.0
Without choroidal caverns	66	31.3	35.7	+ 4.4	0.0065
With choroidal caverns	17	23.8	24.7	+ 0.9	0.83

*Paired t Student

Paired t-tests for comparing CMT pre and post-treatment and findings of uncertain significance revealed a statistically significant decrease in CMT with or without the presence of all biomarkers, with the exception of the presence of choroidal caverns and subretinal pseudocyst (Table 3).

**Table 3:** Univariate analysis compared CMT and findings of uncertain significance

	Eyes per group	Mean basal CMT in μm	Mean CMT after loading dose in μm	Difference in μm	p value*
Without onion sign	51	427.9	367.9	−60	0.04
With onion sign	32	532.5	392.5	−140	0.001
Without prechoroidal cleft	65	469.9	390.3	−79	0.005
With prechoroidal cleft	18	462.2	330.6	−132	0.01
Without intraretinal pseudocyst	51	483.5	392.2	−91	0.01
With intraretinal pseudocyst	32	443.8	353.8	−90	0.003
Without subretinal pseudocyst	67	455	367.8	−87	0.0015
With subretinal pseudocyst	16	523.6	417	−105	0.08
Without choroidal caverns	66	469	372	−97	0.0010
With choroidal caverns	17	465	398.6	−66	0.10

*Paired t Student

## Discussion

Ever since OCT became available, a huge effort has been made to identify OCT biomarkers that facilitate nAMD management and provide solid surrogate variables for treatment response and functional prognosis [17]. Three pathologic changes affecting central retinal morphology have been described in nAMD patients: intraretinal cystoid fluid, subretinal fluid, and pigment epithelial detachment [18, 19]. The most important is the presence of exudative cystoid fluid on OCT as cyst are associated with a higher risk for visual loss associated with fibrosis and atrophy [20].

Despite is initial popularity, functional outcomes correlate poorly with CMT. Solely relying on CMT to make clinical decisions is not recommended [21, 22]. However, CMT gives a first impression of retinal topography [23]. In this study the presence or absence of these findings of uncertain significance show a significatively statistics decrease on CMT, the only exception was the presence of choroidal caverns (no present a statistic significance), this corresponds to what has been described in the literature about their poor correlation.

External limiting membrane (ELM) together with ellipsoid zone (EZ) is considered a criterion that directly reflects photoreceptor function [24]. However, ELM and EZ are no predictor for individual loss or recovery in BCVA, but rather mirrors the current functional state of the retina [25].

Singh et al. realized a literature reviewed OCT-based unique signs finding a worst visual outcome in the presence of outer retinal tubulation and hyperreflective deposits; although not conclusive associated with a worse visual acuity was found in the presence of onion sign, prechoroidal cleft, pseudocyst and choroidal caverns [10].

Pang et al. have shown that onion sign persists even with anti-angiogenic therapy, but there is no description in the literature regarding its improvement in visual acuity [26]. We found that the presence of onion sign was associated with a good anatomical and functional prognosis.

Kim et al. found a poor visual prognosis with presence of prechoroidal cleft, whereas Rahimy et al. showed no influence of clefts on visual acuity [13, 27]. Although not conclusive if clefts can be associated with a worse visual acuity. In our study the absence of prechoroidal was associated significatively with a grater letter gain post-treatment.

Intraretinal pseudocyst can be seen in nAMD eyes with fibroatrophic scars, this suggests that these changes are nonexudative in nature and do not merit any treatment [14]. Hypothetically, these cyst in either nonexudative or exudative AMD may be related to Müller cell degeneration; Querques et al. found that BCVA improved post- antiangiogenic therapy in eyes without degenerative pseudocysts (yet no significantly) and decrease in eyes with pseudocysts [15]. However, subretinal pseudocyst are quite uncommon, they appear as distinct entity compared to intraretinal pseudocyst, and whether these represent nonexudative o exudative process is not clearly understood at present [28]. This study found that absence of pseudocyst (intra and subretinal) was associated with a significatively improve in visual acuity.

Choroidal caverns were hypothesized to form at sites of preexisting choroidal vessels with nonperfused ghost vessels and preserved stromal pillars at level of Haller and Sattler layers [16]. There is limited information on the prognostic significance of caverns [10]. Absence of choroidal caverns in this study was associated significatively with greater letter gain.

Among the limitations of this study are the retrospective analysis, the small sample size, use of only one type of antiangiogenic drug and the time of follow was short (3 months) because a general poor attachment of our population.

## Conclusions

The presence of the following findings was associated with lower visual gains post-treatment: prechoroidal cleft, intraretinal and subretinal pseudocysts and choroidal caverns. This could be attributed to a degenerative change that does not need treatment. Moreover, we found that the onion sign is related as a biomarker of good functional and anatomical prognosis. The onion sign could significate an active CNV that could be beneficiated of treatment, this could be due to chronic exudation trapped in tissue with abundant fibrosis.

OCT biomarkers are suitable to predict VA in patients with nAMD, and to guide the treatment and follow-up of the patients, improving quality of nAMD management. For this reason, continuing to explore new biomarkers that improve the management of nAMD is very important.

## Data Availability

The datasets analyzed during the current study are available from corresponding author on reasonable request.

## Abbreviations

AMD: Age-related macular degeneration
BCVA: Best corrected visual acuity
C: Cortical
CNV: Choroidal neovascularization
CMT: Central macular thickness
ELM: External limiting membrane
ETDRS: Early treatment diabetic retinopathy study
EZ: Ellipsoid zone
LOCS: Lens opacities classification system
nAMD: Neovascular age-related macular degeneration
NC: Nuclear color
NO: Nuclear opalescence
OCT: Optical coherence tomography
P: Posterior subcapsular
RPE: Retinal pigment epithelium
SD-OCT: Spectral domain optical coherence tomography
